# Preoperative hepatic CT perfusion as an early predictor for the recurrence of esophageal squamous cell carcinoma: Initial clinical results

**DOI:** 10.3892/or.2014.2992

**Published:** 2014-01-22

**Authors:** TAKESHI FUJISHIRO, KIYOHIKO SHUTO, KOICHI HAYANO, ASAMI SATOH, TSUGUAKI KONO, GAKU OHIRA, TAKAYUKI TOHMA, HISASHI GUNJI, KAZUO NARUSHIMA, TORU TOCHIGI, TOSHIHARU HANAOKA, SAYAKA ISHII, NORIYUKI YANAGAWA, HISAHIRO MATSUBARA

**Affiliations:** 1 Department of Frontier Surgery, Chiba University Graduate School of Medicine, Chuo-ku, Chiba, Chiba 260-8677, Japan; 2 Department of Surgery, Teikyo University Medical Center, Ichihara, Chiba 299-0111, Japan; 3 Department of Radiological Technology, Chiba University Hospital, Chuo-ku, Chiba, Chiba 260-8677, Japan

**Keywords:** CT perfusion, esophageal cancer, imaging biomarker

## Abstract

Reports suggest that hepatic blood flow may have an association with cancer progression. The aim of the present study was to evaluate whether the hepatic blood flow measured by CT perfusion (CTP) may identify patients at high-risk for postoperative recurrence of esophageal squamous cell carcinoma (ESCC). Prior to surgery, hepatic CTP images were obtained using a 320-row area detector CT. The data were analyzed by a commercially available software based on the dual input maximum slope method, and arterial blood flow (AF, ml/min/100 ml tissue), portal blood flow (PF, ml/min/100 ml tissue) and perfusion index [PI (%) = AF/AF + PF × 100] were measured. These parameters were compared with the pathological stage and outcome of the ESCC patients. Forty-five patients with ESCC were eligible for this study. The median follow-up period was 17 months, and recurrences were observed in 9 patients (20%). The preoperative PI values of the 9 patients with recurrence were significantly higher than those of the 36 patients without recurrence (23.9 vs. 15.9, P=0.0022). Patients were categorized into the following two groups; high PI (>20) and low PI (<20). The recurrence-free survival of the low PI group was significantly better than that of the high PI group (P<0.0001). A multivariate analysis showed that a high PI was an independent risk factor for recurrence (odds ratio, 19.1; P=0.0369). Therefore, the preoperative PI of the liver may be a useful imaging biomarker for predicting the recurrence of patients with esophageal cancer.

## Introduction

Despite improvements in surgical techniques, the recurrence rate after radical resection for esophageal cancer has been reported to reach 36.8–43.4%, and most cases of recurrence occur within two years after surgery (1–8). Establishing a non-invasive and reliable biomarker that enables the early prediction of clinical outcomes is highly desirable.

Previous reports suggest the relationship between tumor progression and hemodynamic changes in hepatic blood flow (9–14). Leveson *et al* (9) reported that gastrointestinal cancer patients with simultaneous liver metastasis exhibited a high hepatic arterial blood flow as measured with scintigraphy when compared with a control group. In colorectal cancer with simultaneous liver metastasis, Leen *et al* (10) demonstrated that the hepatic arterial blood flow was significantly increased and the portal blood flow was significantly decreased in comparison with that observed in healthy volunteers using Doppler ultrasonography. Cuenod *et al* (13) reported that hemodynamic changes, including decreases in the portal blood flow and increases in the mean transit time, may be detected using CT perfusion (CTP) in rats with occult liver metastases. Leggett *et al* (14) reported that the use of CTP in colorectal cancer patients with simultaneous liver metastases revealed that the hepatic arterial blood flow was significantly increased and the portal blood flow was decreased. Therefore, hemodynamic changes in the hepatic blood flow can be a potential biomarker for predicting patient outcomes.

CTP is a non-invasive imaging technique that enables quantification of tissue blood flow in a target organ, by measuring the temporal changes in tissue density following administration of intravenous contrast medium. Since Miles *et al* (15) first described CTP, it has been successfully applied in a variety of clinical conditions of the liver (16–21). In addition, pretreatment CTP has recently been demonstrated to be a useful marker to evaluate therapeutic response or to assess cancer progression or outcome in gastrointestinal cancer patients (22–26).

In this context, our hypothesis is that hepatic blood flow is a more suitable method for assessing cancer progression and outcomes than conventional histopathological and molecular examinations of the primary tumor. Therefore, the aim of this prospective study was to investigate the relationship between the clinical outcomes of esophageal cancer patients and hemodynamic changes in the liver measured by CTP.

## Materials and methods

### Patient population

The present study was approved by the ethics committee of our institution, and informed consent was obtained from all patients. According to the protocol of this study, all patients had clinically and histopathologically proven esophageal squamous cell carcinoma (ESCC) without distant metastasis or other unresectable factors. CTP was performed in all patients prior to surgery. For the patients receiving preoperative treatment, CTP was performed after the completion of the preoperative treatment. The patient eligibility criteria for this study were as follows: (i) 20 to 85 years of age; (ii) no sustained infection with hepatitis virus; (iii) normal liver function; (iv) adequate renal function (a serum creatinine level of <1.5 mg/dl) and (v) no past history of malignant tumors. Sixty-two consecutive patients with ESCC treated at Chiba University Hospital from June 2010 to December 2012 were enrolled in this study. Fifteen patients were excluded since acquired images were not suitable for CTP analysis due to an insufficient concentration of the contrast agent and excessive respiratory movements. Therefore, the data analysis was performed in 47 patients (median age, 67 years; range, 53–82 years). The median follow-up period was 17 months (range, 1–36 months).

### Surgical treatment

Among the study population, 17 patients underwent subtotal esophagectomy with field lymphadenectomy without preoperative chemotherapy. Fifteen patients with a preoperative diagnosis of lymph node metastasis underwent radical resection after two cycles of neoadjuvant chemotherapy. The FP regimen consisted of cisplatin at a dose of 80 mg/m^2^/day via intravenous administration (day 1) and 5-fluorouracil (5FU) at a dose of 800 mg/m^2^/day via continuous intravenous infusion for five days (days 1–5) (27). Four patients who received preoperative chemoradiotherapy (total 40 Gy) and underwent downstaging were included in this study. In addition, 9 patients were treated with endoscopic submucosal dissection (ESD). The pathological tumor stage was assessed using the TNM classification of the Union for International Cancer Control (UICC, 7th edition, 2009).

### Imaging studies

**Figure 1:**
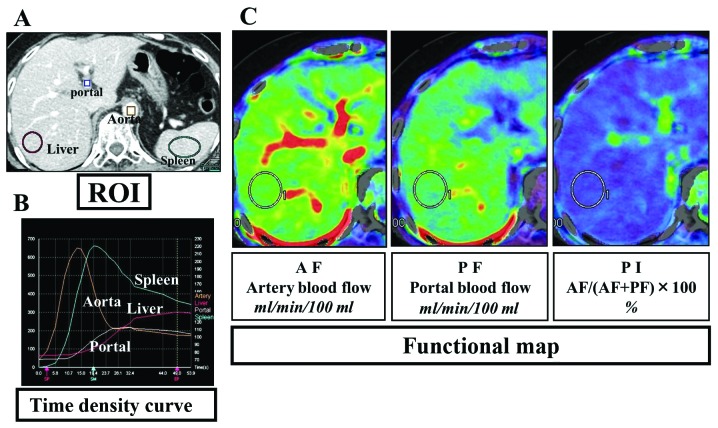
Imaging analysis of hepatic CT perfusion. In order to obtain perfusion parameters, it was necessary to set the region of interest (ROI) in (A) four sites. (B) The time density curve and (C) parameters were calculated and displayed as a functional color map using a software program. A round or oval-shaped ROI was placed on the right hepatic lobe on the functional map, so that the ROI would be as large as possible while avoiding vessels and artifacts.

Hepatic CTP was performed using 320-row area detector CT (Aquilion One; Toshiba Medical Systems, Ohtawara, Japan). First, we performed a non-enhanced CT scan of the upper abdomen to identify the location of the liver. A 16-cm segment of the whole liver (320 sequential 0.5-mm slices) was selected, and a dynamic study of the selected area was performed in the static table position without breath holding. The images were obtained 10 sec after the intravenous injection of 60 ml of iohexol (Omnipaque 300; Daiichi Sankyo, Tokyo, Japan) containing 300 mg of iodine/ml administered at a rate of 6 ml/sec followed by 20 ml of saline chaser using a dual power injector. The scanning parameters were as follows: 0.5-mm reconstructed section thickness, 0.35-sec gantry rotate time, 120 kV, 100 mA. After completion of the perfusion scans, intravenous contrast was administered at 3.5 ml/sec, and a routine thoracoabdominal study was performed. The procedure of the analysis was as follows. First, the raw volume scan data were analyzed using a software program (Body Registration; Toshiba Medical Systems Corporation) in order to remove the influence of breathing motion artifacts. Second, the registered perfusion data were analyzed using a software program (Body Perfusion; Toshiba Medical Systems Corporation), based on the dual input maximum slope method (15,28). The parameters generated by the software included the arterial blood flow (AF, ml/min/100 ml tissue), portal blood flow (PF, ml/min/100 ml tissue) and perfusion index (%) [PI = AF/(AF + PF) × 100]. In order to obtain these parameters, it was necessary to set region of interests (ROIs) in the following four sites; the liver parenchyma, spleen parenchyma, aorta and portal vein. The time density curve (TDC) of the spleen was used to separate the blood flow in the arterial phase and portal phase, and the maximal slope of the liver TDC in each phase was used to calculate both the arterial and portal perfusion. Consequently, a three-dimensional color map (functional map) was displayed (Fig. 1). A round or oval-shaped ROI was placed on the right hepatic lobe on the functional map (29), so that the ROI would be as large as possible while avoiding vessels and artifacts (30). Each parameter was quantified as the average value of all pixels in the ROI. Data were analyzed by gastroenterologic surgeons with at least five years of experience in radiological imaging of gastroenterology. In the comparison of the perfusion parameters by the three readers, there were no significant differences between the readers (data not shown).

### Statistical analysis

Statistical significance was evaluated using the Mann-Whitney U, Kruskal-Wallis and Chi-square tests. In addition, we determined the optimal cut-off value for a high-risk of recurrence using a receiver operating characteristic (ROC) curve. Relapse-free survival curves were drawn according to the Kaplan-Meier method, and the differences between the curves were analyzed by applying the log-rank test. In the multivariate analysis, a logistic regression model was used.

## Results

### Patient characteristics

The pathological stages were as follows: one patient had stage 0; 20 patients had stage I (IA, n=18; IB, n=2); 11 patients had stage II disease (IIA, n=6; IIB, n=5); 12 patients had stage III (IIIA, n=7; IIIB, n=2; IIIC, n=3) and one patient had stage IV disease due to intramural metastasis in the stomach. Data were unavailable for two patients for whom the tumor histologically disappeared under the influence of preoperative treatment. Therefore, data were available for 45 patients in the present study.

### Relationships between the preoperative CTP parameters and histological features

The relationships between the histopathological variables and preoperative perfusion parameters are listed in Table I. The AF and the PI values were significantly higher and the PF values were lower in the patients with poorly differentiated tumors. There were no significant statistical relationships between the pathological stage (TNM classification of UICC) and the perfusion parameters (Table II).

### Correlations between the preoperative CTP parameters and postoperative recurrence

**Figure 2:**
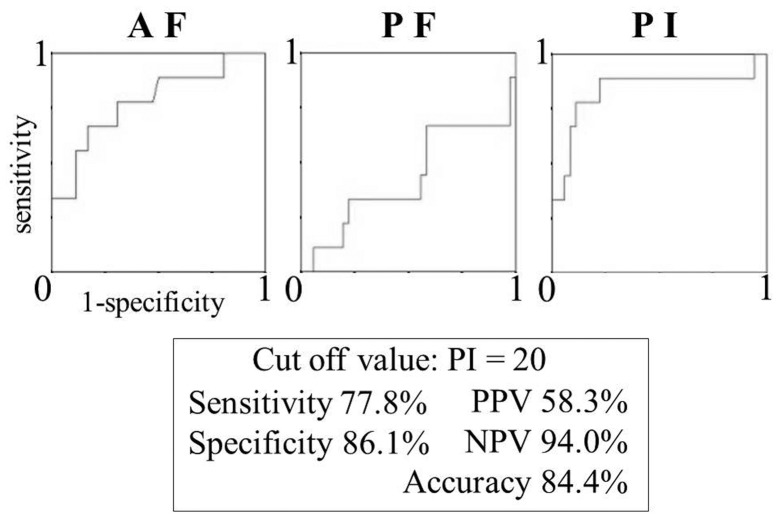
Determination of the cut-off value. We determined the optimal cut-off value for high-risk of recurrence according to the receiver operating characteristic (ROC) curve. AF, arterial blood flow; PF, portal blood flow; PI, perfusion index; PPV, positive predictive value; NPV, negative predictive value.

**Figure 3:**
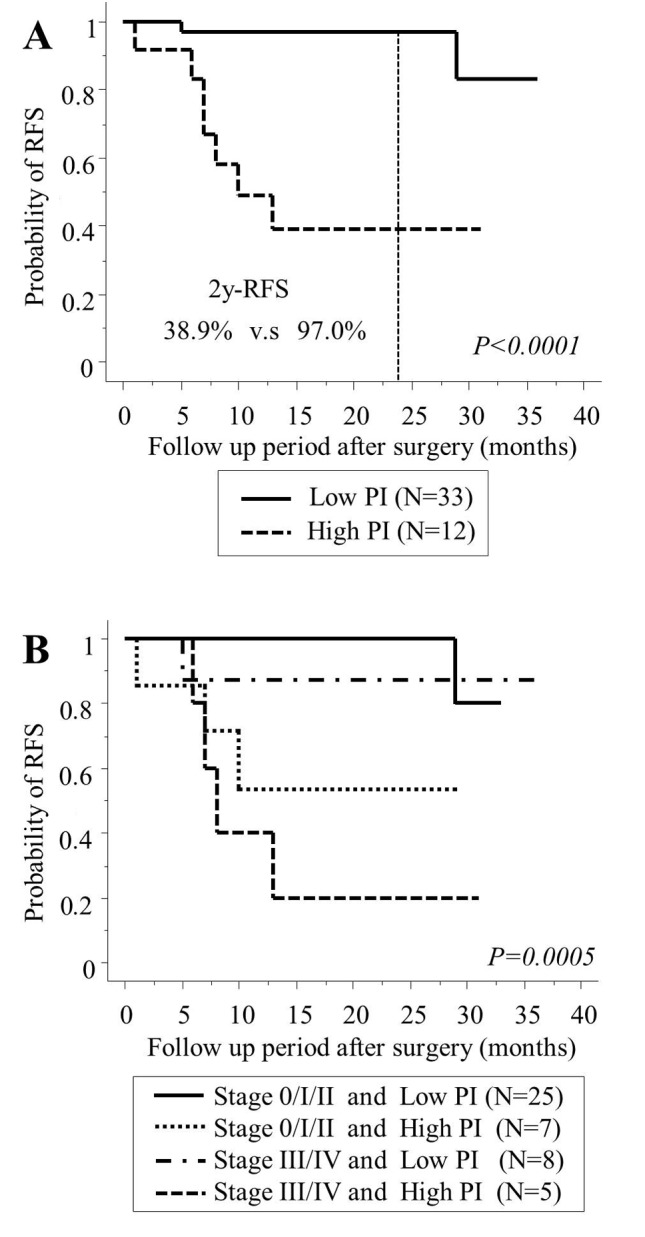
Recurrence-free survival (RFS) curves. (A) An actuarial analysis of the RFS using the Kaplan-Meier method showed that the recurrence rate was significantly higher in the high PI group (PI >20) than that in the low PI group (PI <20) (P<0.0001, log-rank test). (B) In order to prevent the influence of a staging bias, a subgroup analysis was performed. The patients with stage 0/I/II and stage III/IV disease exhibited a similar tendency (P=0.0005, log-rank test).

In the present study, postoperative recurrence was demonstrated in 9 ESCC patients during the short-term observation period by contrast CT. The sites of recurrence in these 9 patients were as follows: liver, 5; lymph nodes, 7; peritoneum, 2; bone, 1; brain, 1; adrenal glands, 1; kidneys, 1 (duplication is included). In the 9 patients with recurrence, AF and PI values were significantly higher than those observed in the 36 patients who did not develop recurrence during the observation period (PI, 23.9 vs. 15.9; P=0.0022, Mann-Whitney U test). The mean PI in the 5 patients with liver metastasis was 26.5 (range, 10.5–42), while the mean PI of the 4 patients with recurrence outside of the liver (site of recurrence: para-aortic lymph node, regional lymph nodes, multifocal hematogenous distant metastases) was 20.8 (range, 17.3–23.3) (P=0.32, Mann-Whitney U test). To determine the optimal cut-off value for predicting patients at a high-risk of postoperative recurrence before surgery, we analyzed the receiver operating characteristic (ROC) curve of the preoperative CTP parameters. We identified the ESCC patients with a PI of >20 as having a high-risk of recurrence and patients with a PI of <20 on preoperative CTP as having a low-risk of recurrence (sensitivity and specificity, 77.8 and 86.1%, respectively) (Fig. 2). We compared the background factors between the two groups and found no significant differences (Table III). An actuarial analysis of the time to recurrence using the Kaplan-Meier method showed that the recurrence rate was significantly higher in the high PI group (>20) than in the low PI group (<20) (P<0.0001, log-rank test) (Fig. 3A). The 2-year recurrence-free survival rate was 38.9% in the high PI group and 97.0% in the low PI group. In order to prevent the influence of a staging bias, a subgroup analysis was performed. The patients with stage 0/I/II and stage III/IV disease exhibited a similar tendency (P=0.0005, log-rank test) (Fig. 3B). A multivariate analysis using the logistic regression model showed that a high PI on preoperative CTP was an independent risk factor for recurrence (odds ratio, 19.1; P=0.0369) (Table IV).

## Discussion

In the present study, we demonstrated a correlation between the preoperative hepatic blood flow and postoperative recurrence. The postoperative recurrence rate was significantly higher in the group with increased preoperative PI values. Similar observations have been reported in previous studies in gastrointestinal cancer (9,31–33). Huguier *et al* (31) reported that the preoperative hepatic perfusion scintigraphy in patients with gastrointestinal cancer (including esophageal, gastric, colorectal and pancreatic cancer) was useful for identifying patients at low-risk for recurrence of liver metastasis and for avoiding unnecessary adjuvant chemotherapy. In patients with colorectal cancer, Warren *et al* (32) reported similar results in a prospective assessment of a liver perfusion analysis using scintigraphy. Furthermore, it has been reported that an increased ratio of hepatic arterial blood flow to total liver blood flow detected on color duplex Doppler ultrasonography is significantly associated with a high incidence of postoperative liver metastasis and a poorer prognosis regarding colorectal cancer (33). These reports, which demonstrate that the hepatic blood flow is a useful marker for assessing the biological malignancy of the tumor and predicting the outcome, support our present observations. However, most of these reports on hepatic blood flow focused on colorectal cancer, and there are few reports on esophageal cancer. Considering the high early recurrence rate of postoperative esophageal cancer (1,2), liver perfusion analysis may offer an ideal biomarker that can provide an optimal follow-up strategy for postoperative esophageal cancer patients.

The underlying mechanisms of hepatic hemodynamic changes have been discussed in previous reports (10–12,32,34–39). Evidence obtained from a liver metastasis model in rats suggests that a circulating vasoconstrictor is responsible for the increased splanchnic vascular resistance and subsequent reduction in portal venous flow (10,11,34). This reduction in portal venous flow may lead to a relative increase in the arterial flow in the liver (11,12,40,41). This hypothesis is known as hepatic arterial buffer response (HABR), noted under various experimental conditions such as endotoxinemia (42) or experimental portal vein ligation (43), in the clinical setting after liver transplantation (44) and in patients with advanced cirrhosis (45–48). HABR is an intrinsic regulatory mechanism of the liver to maintain total hepatic blood flow when portal perfusion decreases. According to this hypothesis, since arterial blood is increased when the portal blood is decreased due to the progression of the tumor, we considered that PI provided by the ratio of these parameters may be an ideal marker with high sensitivity. Its potential has been demonstrated by the present study.

Our study is associated with a few limitations. First, our findings are based on single center data, and the sample size was small. Therefore, there may be a selection bias. Our findings must be confirmed in multicenter investigations, and a larger patient population should be studied. Second, a consensus and standardization of data acquisition and analysis of methods (including the optimal positioning of the ROI on the liver) have yet to be established for CTP. Third, we did not perform any pathological validation studies comparing CTP parameters with more established markers of angiogenesis, such as microvessel density or the level of vascular endothelial growth factor.

In conclusion, we showed that the preoperative hepatic blood flow measured by CTP may be a valuable biomarker for predicting the early recurrence of patients with esophageal squamous cell carcinoma. To obtain more conclusive results, a larger patient population should be studied. Nevertheless, our results provide important insight into selecting the optimal therapeutic strategy for the treatment of esophageal squamous cell carcinoma.

## Figures and Tables

**Table I:** Preoperative hepatic perfusion parameters and clinicopathological features of the patients with ESCC.

Variables	AF	PF	PI
Tumor location			
Upper	28.5	128.4	18.5
Lower	25.7	127.1	16.9
P (or yp) stage			
0/1	25.8	128.3	17.0
2/3/4	27.7	127.1	18.0
P (or yp) T			
0/1	26.0	130.8	16.8
2/3	27.6	127.7	18.2
P (or yp) N			
N0	26.4	129.1	17.1
N1/2/3	27.5	128.4	18.1
Differentiation^a^			
Well/moderate	24.9	128.0	16.4
Poor	34.1	119.0	22.7

a P<0.05 (Mann-Whitney U test). AF and PF are expressed in ml/min/100 ml tissue. AF, arterial blood flow; PF, portal blood flow; PI, perfusion index (%).

**Table II:** Relationships between pathological stage and the preoperative perfusion parameters of the ESCC patients.

P stage (UICC)	AF	PF	PI
0 (n=1)	16.8	127.3	11.7
IA/B (n=18/2)	26.2	128.3	17.2
IIA/B (n=6/5)	27.9	133.1	17.6
IIIA/B/C (n=7/2/3)	25.8	124.9	17.3
IV (n=1)	53.8	119	31.1
P-value^a^	N.S	N.S	N.S

a Kruskal-Wallis test. AF and PF are expressed in ml/min/100 ml tissue. UICC, Union for International Cancer Control. AF, arterial blood flow; PF, portal blood flow; PI, perfusion index (%).

**Table III:** Background of the high-risk and low-risk recurrence groups according to the preoperative PI values.

Variable	Categories	High PI (n=12)	Low PI (n=33)
Mean age (years)	-	69.3	66.4
P (or yp) stage	0/I/II/III/IV	0/3/4/4/1	1/17/7/8/0
P (or yp) T	0/1/2/3/4	0/3/2/7/0	1/18/5/9/0
P (or yp) N	0/1/2/3	6/2/1/3	21/8/4/0
Preoperative therapy, n (%)	-	6 (50%)	13 (39.4%)
Complication^b^ (Clavien-Dindo)	Non, I, II/III-V	10/2	26/7
Grade	0/1/2/3	0/5/1/0	2/7/3/1
Recurrence rate, n (%)	-	7 (58.3%)^a^	2 (6.1%)^a^

a P<0.01 (Chi-square test).

b All complications were included that occurred in the perioperative period regardless of the direct effect of the surgical procedure.

**Table IV:** Univariate and multivariate analyses of the prognostic parameters for time to recurrence after surgery in the esophageal cancer patients.

		Univariate			Multivariate		
			
Variable	Categories	Odds ratio	95% CI	P-value	Odds ratio	95% CI	P-value
PI	≥20 vs. <20	21.7	3.5–135.8	0.0010	19.1	1.2–303.7	0.0369
P (or yp) T	2/3/4 vs. 0/1	11.2	1.3–99.3	0.0300	14.2	0.323–621.7	0.1693
P (or yp) N	+ vs. −	4	0.8–18.8	0.0795	1.0	0.023–48.2	0.9806
P (or yp) stage	III/IV vs. 0/I/II	4.4	0.9–20.2	0.0589	2.5	0.066–91.5	0.6253
Differentiation	Poor vs. mod/well	4.5	0.9–22.7	0.0701	5.5	0.316–94.6	0.2431
Location	Upper vs. lower	1.3	0.3–5.5	0.7612	2.8	0.236–33.4	0.4145
Age (years)	≥65 vs. <64	1.4	0.3–6.6	0.6491	0.381	0.013–11.0	0.5740

CI, confidence interval; PI, perfusion index.
